# A reduction in public funding for fertility treatment - an econometric analysis of access to treatment and savings to government

**DOI:** 10.1186/1472-6963-12-142

**Published:** 2012-06-08

**Authors:** Georgina M Chambers, Van Phuong Hoang, Rong Zhu, Peter J Illingworth

**Affiliations:** 1 Perinatal and Reproductive Epidemiology Research Unit, The University of New South Wales, Level 2, McNevin Dickson Building, Randwick Hospitals Campus, Sydney, 2031, Australia; 2 National Institute of Labour Studies, Flinders University, Faculty of Social and Behavioural Sciences, Flinders University, GPO Box 2100, Adelaide, 5001, Australia; 3 IVFAustralia Pty Ltd, 176 Pacific Highway, Greenwich, 2065, Australia

**Keywords:** Assisted reproductive technology, *In vitro* fertilization, Infertility, Policy evaluation, Econometrics

## Abstract

**Background:**

Almost all assisted reproductive technology (ART) and intrauterine insemination (IUI) treatments performed in Australia are subsidized through the Australian Government’s universal insurance scheme, Medicare. In 2010 restrictions on the amount Medicare paid in benefits for these treatments were introduced, increasing patient out-of-pocket payments for fresh and frozen embryo ART cycles and IUI. The aim of this study was to evaluate the impact of the policy on access to treatment, savings in Medicare benefits and the number of ART conceived children not born.

**Methods:**

Pooled quarterly cross-sectional Medicare data from 2007 and 2011 where used to construct a series of Ordinary Least Squares (OLS) regression models to evaluate the impact of the policy on access to treatment by women of different ages. Government savings in the 12 months after the policy was calculated as the difference between the predicted and observed Medicare benefits paid.

**Results:**

After controlling for underlying time trends and unobserved factors the policy change reduced the number of fresh embryo cycles by almost 8600 cycles over 12 months (a 16% reduction in cycles, p < 0.001). The policy effect was greatest on women aged 40 years and older (38% reduction in cycles, p < 0.001). Younger women engaged in relatively more anticipatory behaviour by bringing forward their fresh cycles to 2009. Frozen embryo cycles, which are approximately one quarter of the cost of a fresh cycle, were only marginally impacted by the policy. Utilisation of IUI cycles were not impacted by the policy. After adjusting for anticipatory behaviour, $76 million in Medicare benefits was saved in the 12 months after the policy change (0.47% of annual Medicare benefits). Between 1200 and 1500 ART conceived children were not born in 2010 as a consequence of the policy.

**Conclusions:**

The introduction of the policy resulted in a significant reduction in fresh ART cycles in the first 15 months after its introduction. Further evaluation on the long term impact of the policy with regard access to treatment and on clinical practice, particularly the number of embryos transferred, is crucial to ensuring equitable access to fertility treatment and the health and welfare of ART children.

## Background

### Infertility and its treatment

Infertility affects approximately 10% of couples at any given time worldwide, representing significant personal suffering to millions of couples around the globe. Based on recent estimates, over 72 million women worldwide are currently infertile, of which approximately 40 million will seek healthcare assistance [1]. Fertility treatments range from advice about how to optimise natural conception to complex and invasive medical interventions including surgery and assisted reproductive technologies (ART). ARTs are generally considered to include all treatments or procedures that include the *in vitro* handling of human oocytes [eggs] and sperm or embryos for the purposes of establishing a pregnancy [2]. The most common ART is *in vitro* fertilisation (IVF). A typically ART ‘cycle’ involves the stimulation of a woman’s ovaries with hormones to produce multiple mature eggs, removal of the mature eggs under anaesthesia, fertilisation of the eggs outside of the body to create embryos, and the transfer of one or more 2-6 day-old embryos back into the woman’s uterus. This type of ART cycle is termed a fresh embryo transfer cycle (Fresh Cycle). Excess embryos can be frozen and transferred back into the woman at a later date in what is termed a frozen embryo transfer cycle (Frozen Cycle). Around 20% of ART cycles result the birth of a live born child. However, the type of cycle (e.g. Fresh or Frozen), female age, number of previous treatment attempts and type of infertility all contribute to the likelihood of success [3-5]. Since the birth of the first ART baby - Louise Brown in England in 1978 - over 4.7 million babies have been born worldwide from ART treatments. The use of ART treatment is increasing by 5-10% per annum in most countries with the worldwide activity estimated to be 1.5 million cycles per year [6]. While access and demand for ART treatment varies significantly between countries - especially between developed and developing countries - up to 5% of children in some countries are now conceived through ART treatments [3]. ARTs are the most advanced fertility treatments available and because of high medical, scientific, pharmaceutical and staff costs are also the most expensive, with the cost of a Fresh Cycle ranging from $12,500 US dollars (USD) in the US to $4000 USD in Japan. Because a Frozen Cycle only requires the transfer of a previously created embryo into a women’s uterus, costs are generally one quarter of a Fresh Cycle [7]. Multiple Fresh and Frozen Cycles may be needed to achieve a pregnancy and many couples will discontinue treatment after multiple failed attempts because of the financial, emotional or physical burden of treatment. Along with ARTs the most common form of medical intervention to treat infertility is intrauterine inseminaton (IUI), whereby sperm - a partner’s or donor’s - is deposited directly into a woman’s uterus to aid conception. This can be either performed in a treatment cycle where the ovaries have been stimulated with hormones to mature multiple eggs or in an unstimulated cycle.

### Funding arrangement for fertility treatment

**Figure 1:**
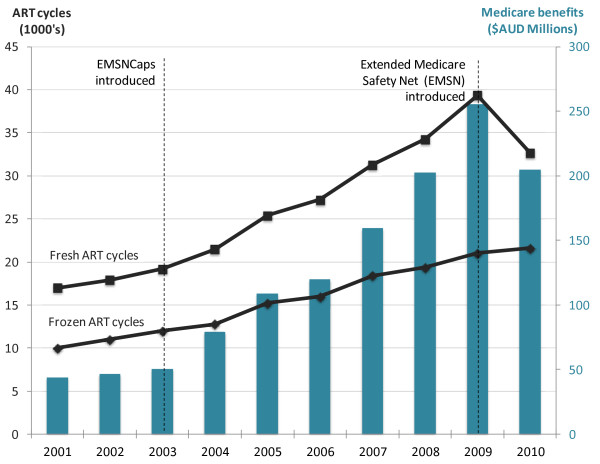
**ART treatment cycles and Medicare Benefits Scheme benefits, Australia 2011-2010.** Source: Medicare online statistics: http://www.medicareaustralia.gov.au/provider/medicare/mbs.jsp#N10030.

Funding arrangements for ART and IUI vary substantially across jurisdictions, countries and time, ranging from almost unrestricted funding in Israel to no public funding in the US, South America and most developing countries. [7,8]. Australia has had a tradition of supportive public funding of ART and IUI treatment through its universal healthcare insurance system, Medicare. Since 2000, women have been eligible for partial reimbursement of all ‘medically necessary’ ART and IUI cycles regardless of age or numbers of previous cycles and children. The most significant policy change to affect funding of ART in the last decade was the introduction of the Extended Medicare Safety Net (EMSN) in 2004. This policy is applicable to all out-patient Medicare services and was introduced to assist patients with high out-of-pocket expenses. The EMSN reimburses 80% of future out-of-pocket expenses for all out-patient Medicare services once an annual threshold is reached. This scheme had a significant impact of ART and IUI treatments because they are high cost treatments mostly undertaken in an out-patient setting. With the average cost of a Fresh Cycle in Australia being about $8000, Australian dollars (AUD) most patients reached their out-of-pocket threshold after just one treatment cycle, making additional ART or IUI cycles in the calendar qualify for EMSN benefits. The EMSN policy effectively reduced patient out-of-pocket expenses for a Fresh Cycle from approximately $4000 to $1500. Primarily because of this, utilisation of ART treatments increased by 72% over five years from 31,200 cycles in 2003 to 53,600 cycles in 2008. The benefits paid by Medicare increased by over 300% from $50.0 M to $202.2 M over the same period because of the increase in utilisation and the increase in benefits paid per cycle under the EMSN scheme (Figure 1).

A review of the EMSN scheme in 2006, found that ART treatment comprised 22% of all benefits paid by the EMSN scheme. It concluded that due to the unlimited nature of the benefits available through the EMSN scheme some of the 68 private fertility clinics in Australia opportunistically raised their fees in the knowledge that the majority of the cost would be funded by government - this meant that patients did not fully see the reduction in out-of-pocket expenses that the EMSN scheme had been designed to support [9]. In response to these findings, ‘caps’ on the amount of EMSN benefits that could be paid for ART and IUI treatment were announced in October 2009 and implemented in January 2010. In effect this meant that if the fees charged by fertility clinics remained unchanged, patient would need to pay more for their treatment while the government would pay less. Out-of-pocket expenses increased for Fresh Cycles by $500-$1000 to ~ $2000-$2500 per cycle, for Frozen Cycles by $300-$500 to ~ $1000 per cycle and for IUI Cycles by $300-$500 to ~ $600 per cycle. The changes to the EMSN scheme in relation to fertility treatment was expected to provide savings of $69.4 million annually [10].

The aims of this study are to evaluate the impact of the EMSN ‘caps’ (hereon referred to as EMSNCaps) on:

i. Access ART treatment (Fresh and Frozen Cycles) and IUI Cycles by different age groups of women seeking treatment.

ii. Savings achieved by the Australian Government in Medicare benefits from the EMSNCap policy with respect to ART and IUI treatment.

iii. The reduction in the number of ART and IUI infants not born in the first year after the EMSNCap (2010) due to the increases in the out-of-pocket expenses.

## Methods

### Data sources

Pooled cross-sectional data on ART and IUI services undertaken between January 2007 and June 2011 were obtained from the Australian Government’s Medicare Information Service Branch. The data were extracted from a national dataset of all services for which Medicare benefits are paid (estimated to be 100% of Fresh and Frozen Cycles performed in Australia). The data included counts of persons and services, average provider fees and Medicare benefits. To evaluate the impact of the EMSNCaps on ART and IUI access by female age (<32, 32-33, 34-35, 36-37, 38-39, 40-41, 42 + years) Medicare provided quarterly averages of the provider fees charged, Medicare benefits paid and out-of-pocket expenses for the different cycle types. Medicare Australia supplied these data for nine ART Medicare Benefits Schedule (MBS) items before the Caps and eleven MBS items after the Caps; 13200 (fresh embryo ART cycle), 13201(subsequent fresh embryo ART cycle in a calendar year, introduced in 2010), 13202 (cancelled ART treatment before oocyte retrieval, introduced in 2010), 13203 (ovulation induction for IUI), 13209 (planning and management of ART or IUI), 13212 (oocyte retrieval), 13215 (fresh embryo transfer before 2010 and fresh or frozen embryo transfer after 2010), 13218 (frozen embryo transfer cycle), 13221 (semen preparation), 13251 (intracytoplasmic sperm injection). A combination of these MBS items are used to bill Medicare for ART and IUI treatment and represent the type and sequential steps in each type of treatment cycle. For example, an initial Fresh Cycle performed in a calendar year after 2010 would typically incur Medicare MBS items 13209, 13200, 13201, 13215, 13218 (Table 1). The age-stratified data represented 84 observations before the caps and 42 observations after the caps. However, because not all claims had been lodged with Medicare at the time of the data extraction, estimates pertaining to utilisation rates for the second quarter of 2011 were excluded.

**Table 1:** Medicare Benefits Schedule (MBS) Assisted Reproductive Technology (ART) billing items

**Cycle type**	**Before January 2010**	**After January 2010**
	**(pre EMSNCap policy)**	**(post EMSNCap policy)**
Fresh Cycle (uniquely identified by items 13200 and 13201)	13209, 13200, 13212, 13215, 13221, 13251	13209, 13200, 13212, 13201, 13215, 13251
Frozen Cycle (uniquely identified by item 13218)	13209, 13218	13209,13215,13218
IUI Cycle (identified by item 13203)	13209, 13203, 13221	13209, 13203, 13221

Notes:

13209: This item is assigned to all ART treatment types. Before 2010 it was typical for providers to assign a much higher fee to Fresh Cycles than either Frozen Cycles of IUI. Based on a survey of ART provider charging practices in 2007, the average fees, MBS benefits and OOPs were assigned to Frozen Cycles and IUI Cycles were 34% of the weighted average fee of 13209 assigned to a Fresh cycle, by quarter and age group.

13251: This item is used to bill intracytoplasmic sperm injection which is a laboratory technique to aid fertilisation. The average fees, Medicare benefits, OOPs, and number of ICSI services and patients were assigned to each quarter and age group based on the proportion of 13200 and 13201 cycles attracting a 13251 item.

13203: Prior to 2010, the MBS item 13203 was assigned to both cancelled Fresh Cycles and IUI cycles. After 2010, a specific MBS item was created for cancelled Fresh Cycles (13202) and item 13203 was retained for IUI. Therefore to estimate utilisation for IUI cycles before the 2010, the age-specific proportion of 13202 to 13203 items performed in 2010 was used to adjust service and patient counts for MBS item 13203.

Because multiple MBS items represent an ART cycle, the analysis was undertaken based on an ‘episode of care’ for undertaking a Fresh Cycle, Frozen Cycle or IUI Cycle. The steps involved in preparing the MBS item-based data to represent a treatment cycle involved the following steps for each age group, time period and cycle type.

1. Dollar figures for provider fees and Medicare benefits were converted to constant 2010 dollars using the ABS Health Price Index [11].

2. Provider fees and Medicare benefits for each MBS item were calculated as the total average fee and benefits divided by the no of services.

3. Out-of-pocket expenses were calculated as the average provider fee minus the average Medicare benefit.

4. The average cycle-based provider fees, benefits and out-of-pocket expenses for each of the three cycle types were calculated by summing weighted averages for each of the MBS items associated with a Fresh, Frozen and IUI cycle (Table 1).

5. MBS Items 13200 and 13201 were used to uniquely identify utilisation of Fresh Cycles and MBS Item 13218 was used to uniquely identify Frozen Cycles. MBS Item 13203 was used to identify IUI utilisation, however an adjustment was made to account for MBS Item 13203 also being used to bill cancelled ART cycles before 2010. 

Ethics approval for this project was obtained from The University of New South Wales, Human Research Ethics Committee.

### Empirical analysis

#### The effect of the EMSNCap policy on women in different age categories

A series of Ordinary Least Squares (OLS) regression models were used to evaluate whether the introduction of EMSNCaps was associated with significant changes in access to Fresh, Frozen and IUI Cycles by women of different ages. The quarterly pooled cross-sections of Medicare data stratified by age between January 2007 and June 2011 were used to construct the following model.

(1) $$y_{\mathrm{ita}}=\beta_{0}+\beta_{1}EMSNCap+\beta_{2}Timetrend_{t=1,\ldots ,18}+\beta_{3}Seasonal\;dummy+\beta_{4}y_{-1}+\in_{\mathrm{ita}}$$

In essence the time-trend was compared for each of the three cycle types before and after the EMSNCap was introduced in 2010. The two dependent variables for each cycle type were (i) number of cycles, and (ii) number of patients for cycle type *i* in quarter *t* and age *a* (reported by age groups ≤33, 34-39 and ≥40 years). A linear ordinal time-trend independent variable (Timetrend) was included to capture underlying trends in the data and quarterly seasonal dummies (Seasonal dummy) were included to control for the highly seasonal nature of the data. To capture the *ceterus paribus* impact of the policy change, a step dummy variable (EMSNCap) was used to impose a permanent shift in the level of the series, taking the value of 0 before 2010 and 1 after 2010. The dependent variable was lagged by one time period for each age group to take account of inertia inherent in patients’ decisions to undertake fertility treatment, existing capacity of fertility clinics, and age-specific unobserved factors. The estimated coefficient of the lag of the dependent variable was statistically significant for all models. Further, the R square statistic was greater for all age-specific models with lag compared to those without indicating better fitted estimates.

### Anticipatory behaviour

The introduction of the EMSNCap policy was announced in October 2009, but not implemented until January 2010. It was evident from the service counts that patients brought forward their ART treatment to 2009 in anticipation of the policy change, in effect having their treatment before the ‘price rise’. To account for this anticipatory behaviour, the average percentage change from the third quarter to the fourth quarter in 2007 and 2008 was used to adjust the service and patient counts for each age group in the fourth quarter in 2009 and the first quarter in 2010. This adjustment reduced the number of cycles undertaken in the fourth quarter in 2009 and increased them in the first quarter of 2010, in effect removing the anticipatory behaviour from the data. Results are reported with and without the adjustment of anticipatory behaviour.

### Distribution of the number of cycles in each year

To determine if the number of cycles undertaken by an individual women in a calendar year have changed in response to the EMSNCap policy, data on the distribution of patients undergoing 1, 2, 3, and ≥4 Fresh and Frozen Cycles was obtained. A chi squared statistic was used to test for differences in the annual proportion of cycles undertaken by women before the EMSNCap (2007-2009) with the first year after the EMSNCap (2010).

### Medicare benefit savings and number of cycles not performed due to policy

To estimate the savings to the Australian Government in Medicare benefits and the number of cycles not undertaken in the first year after EMSNCaps were introduced (2010), we calculated the difference between the predicted values of these two dependent variables (assuming the EMSNCap policy had not been introduced) from the observed values for the dependent variables. To do this, we regressed the average Medicare benefits and number of cycles using the age-stratified data before 2010 (84 observations from 2007 to 2009), and used the resulting coefficients to predict the Medicare benefits and number of cycles for the periods after 2010 (42 observations from quarter 1, 2010 to quarter 2, 2011). The estimates were obtained using Equation (1) with and without adjustment for anticipatory behaviour. When estimating the co-efficients for Medicare benefits, the lag of the dependent variable was omitted because the EMSNCaps had an instantaneous effect of the amount Medicare paid in benefits for fertility treatment.

The savings in Medicare benefits to the Australian Government in 2010 were due to both (i) fewer cycles being undertaken after the EMSNCap policy and (ii) the cycles that were undertaken attracting less Medicare benefits. Therefore to calculate the total savings in Medicare benefits, we summed the following values for each cycle type where the EMSNCap was shown to significantly affect access:

1. Cycles not undertaken in 2010; calculated by multiplying the number of cycles forgone by the average predicted Medicare benefits that would have been paid had the EMSNCaps policy not been introduced.

2. Cycles undertaken in 2010; calculated by multiplying the observed number of cycles undertaken in 2010 by the difference in the predicted and observed Medicare benefits per cycle.

The number of live-births (defined as the delivery of at least one live-born infant of at least 20 weeks gestation) that would have been born had the EMSNCap policy not been introduced was calculated by multiplying the number of cycles foregone by the live-birth in Australia in 2009. Because the price of ART treatment is higher after the EMSNCap, this may create a financial incentive to transfer higher numbers of embryos during treatment. Therefore to allow for a possible increase in the ART multiple birth rate after the EMSNCap policy, the number of live born babies was calculated using the multiple birth rate from 2007 (10.2%) which is 2 percentage points higher than in 2009 [4].

## Results

### Descriptive statistics

#### Age specific groups, provider fees, Medicare benefits and out-of-pocket costs

**Figure 2:**
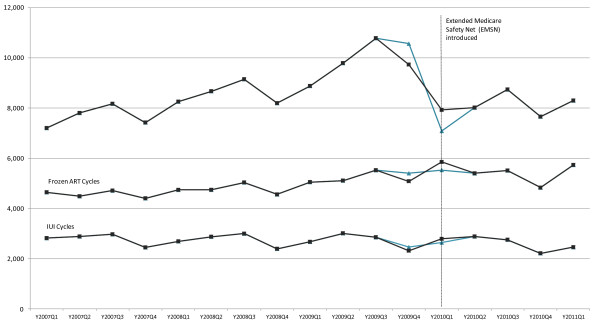
**Number of treatment cycles, all ages Australia 2007-2011.** Source: Medicare service data provided by the Medicare Information Service Branch.

Prior to the introduction of the EMSNCaps there was an annual increase in Fresh Cycles of +12% and +17% in 2008 and 2009 respectively. However with the introduction of the EMSNCaps, the number of Fresh Cycles performed in 2010 decreased by - 21% from 40,017 in 2009 to 31,504 in 2010. After adjusting for anticipatory behaviour the annual decrease in Fresh Cycles in 2010 was - 16% (32,339). Compared with Fresh Cycles, the annual increase in Frozen Cycles before the EMSNCaps followed by the decrease after the policy was less pronounced. There was a 5% and 9% annual increase in Frozen Cycles in 2008 (19,085) and 2009 (21095), followed by a 1% increase in cycles in 2010 (21,276). IUI Cycle utilisation remained relatively unchanged before the EMSNCaps at approximately 11,000 cycles in 2008 and 2009, followed by a decrease of 5% in 2010 (Table 2 and Figure 2).

**Table 2:** Summary statistics for fertility treatments, Medicare Australia data, 2007-2011

	**2007 Q1 to 2009 Q4**					**2010 Q1 to 2011Q2**				
	**Pre EMSNCap**					**Post EMSNCap**				
**Fresh Cycles**	Obs	Mean	SD	Min	Max	Obs	Mean	SD	Min	Max
No of Patients	84	1166	265	781	2130	35	1117	237	750	1831
Number of Cycles	84	1249	278	810	2241	35	1137	237	751	1856
Average provider fees	84	7393	475	6182	8187	42	7664	172	7355	8014
Average Medicare benefits	84	5333	548	4101	6244	42	5016	189	4744	5421
Average out-of- pockets	84	2059	188	1671	2551	42	2648	82	2525	2909
**Frozen Cycles**	Obs	Mean	SD	Min	Max	Obs	Mean	SD	Min	Max
No of Patients	84	594	147	337	1074	35	654	140	428	1040
Number of Services	84	696	178	383	1248	35	772	171	496	1255
Average Fees	84	2097	106	1827	2373	42	2367	89	2208	2731
Average Benefits	84	1447	153	1121	1756	42	1323	71	1210	1427
Average OOP	84	650	94	483	858	42	1044	95	935	1317
**IUI Cycles**	Obs	Mean	SD	Min	Max	Obs	Mean	SD	Min	Max
No of Patients	84	264	155	84	674	35	265	136	119	641
Number of Services	84	394	229	127	1015	35	370	193	158	923
Average Fees	84	1381	159	1102	1786	42	1195	66	1064	1386
Average Benefits	84	1007	151	731	1372	42	542	21	505	580
Average OOP	84	374	59	292	547	42	653	62	546	849

All dollar figures in constant 2010 Australian dollars. Cycle costs do not include pharmaceuticals and hormones. OOP denotes patient out-of-pocket expenses.

### Econometric results

#### Access to fresh cycles by age

Equation (1) was estimated to describe the effect of the EMSNCap policy on three clinically relevant age groups (≤33 years, 34-37 years, ≥38 years) and all ages combined (Table 3). The estimated results show that the coefficient on EMSNCap policy was negative and statistically significant at 1% across all age groups, except for age group ≤33 (10% significance). Overall, the number of Fresh Cycles decreases by 307 cycles per quarter for all age groups in the 15 months after the EMSNCap, a decrease of 8596 cycles over 12 months. Specifically, the number of services decreases by 430 and 497 cycles per quarter in the 34-39 years and ≥40 years, respectively (p < 0.001), and 305 cycles per quarter in women aged ≤33 years (p < 0.1). After adjusting for anticipatory behaviour, the number of Fresh Cycles decreased by 195 cycles per quarter across all age groups (5460 Fresh Cycles over 12 months). Interestingly the magnitude of the policy effect was less in the ≤33 years (183 Fresh Cycles) and 34-39 years (276 Fresh Cycles) age groups than for women aged ≥40 years (437 Fresh Cycles), indicating the women in the younger age groups engaged in relatively more anticipatory behaviour by bringing their treatment forward to 2009. This may be a reflection of the price pressures created by the EMSNCaps being greater for the younger age groups.

**Table 3:** Determinants of number of Fresh Cycles undertaken

	**Base model and lag**				**With anticipatory behaviour and lag**			
				**all age**				**all age**
**Variable**	**Age ≤ 33**	**34 ≤ Age ≥ 39**	**Age ≥ 40**	**groups**	**Age ≤ 33**	**34 ≤ Age ≥ 39**	**Age ≥ 40**	**groups**
**EMSNCap effect**	-305.83*	-429.52***	-497.31***	-307.04***	-183.17*	-275.63***	-436.91***	-195.24***
	143.84	59.82	56.09	72.58	80.23	40.70	63.83	48.20
Lag of D. Variable	0.88***	0.32**	-0.07	0.70***	0.95***	0.56***	0.001	0.82***
	0.13	0.10	0.10	0.11	0.06	0.07	0.12	0.06
Season 1 (base)								
Season 2	335.00**	132.34**	-21.77	123.40**	230.57***	81.25**	-45.02	48.77
	94.08	38.29	34.23	45.64	54.22	28.25	36.58	35.29
Season 3	345.06**	158.65***	-76.69*	117.40*	279.29***	112.51***	-95.47**	66.87
	96.6512	34.5121	31.1946	47.48	62.4833	25.8843	33.7326	38.5072
Season 4	62.98	-18.28	-236.29***	-79.41	-42.13	-108.45***	-262.41***	-159.02***
	90.82	38.17	33.58	42.29	47.57	27.11	35.10	31.69
Time Trend	26.38*	35.28***	51.50***	25.45***	14.03	20.94**	45.57***	14.29**
	11.98	4.10	5.70	6.30	7.77	3.61	6.59	4.32
Constant	-178.31***	576.37***	963.39***	183.49	-122.03	416.99***	934.94***	168.30*
	126.19	107.59	95.43	110.00	103.93	71.97	100.54	75.76
N	32	48	32	112	32	48	32	112
df	6	6	6	6	6	6	6	6
R squared	0.85	0.81	0.89	0.71	0.94	0.88	0.89	0.81

OLS Regression method is used. Estimated coefficients and standard errors are presented. df denotes the degrees of freedom. * Significance at 10%, ** Significance at 5%, *** Significance at 1%. Each column reports a separate model specification.

Estimates of the semi-log of Equation (1) show that after accounting for anticipatory behaviour, the EMSNCap policy caused a 16% decrease in the Fresh Cycles across all ages, 14% for women aged ≤34 years, 21% for woman aged 34-39 years and 38% for women aged ≥40 years (Table 4).

**Table 4:** Semi-elasticity of number of services and number of patients after the introduction of EMSNCap Policy (for Fresh, Frozen and IUI Cycles)

	**Base model and lag**				**With anticipatory behaviour and lag**			
				**all age**				**all age**
**Variable**	**Age ≤ 33**	**34 ≤ Age ≥ 39**	**Age ≥ 40**	**groups**	**Age ≤ 33**	**34 ≤ Age ≥ 39**	**Age ≥ 40**	**groups**
	**Fresh Cycles**							
Number of services	-0.22*	-0.34***	-0.44***	-0.25***	-0.14*	-0.21***	-0.38***	-0.16***
Number of patients	-0.19*	-0.28***	-0.32***	-0.20***	-0.12*	-0.16***	-0.28***	-0.13***
	**Frozen Cycles**							
Number of services	-0.11*	-0.07*	-0.09	-0.09**	-0.06	-0.08	0.07	-0.04
Number of patients	-0.11*	-0.07*	-0.10	-0.09**	-0.07	-0.09*	0.06	-0.05
	**IUI Cycles**							
Number of services	-0.14**	-0.07	0.13	-0.01	-0.04	-0.06	0.12	0.00
Number of patients	-0.04	-0.04	0.15	0.02	-0.03	-0.04	0.16*	0.02

OLS Regression method is used for the semi-elasticity models. Estimated coefficients are presented. * Significance at 10%, ** Significance at 5%, *** Significance at 1%. Each column reports a separate model specification.

Because a fertility patient may undergo a number of Fresh Cycles it is important to investigate if the EMSNCap policy decreased not only the number of Fresh Cycles, but also the number of patients accessing treatment. Estimates using Equation (1) were used for this purpose and reported in Table 5. Overall the number of women accessing Fresh Cycles decreased by 233 patients per quarter (6524 women over 12 months). The age specific trends were similar to those for service counts; the greatest impact of the policy was in older age groups, while younger women engaged in more anticipatory behaviour. The semi-log of Equation (1) for number of patients shows that after accounting for anticipatory behaviour there was a decrease in the number of patients undergoing Fresh ART Cycles of 13% for all ages, 12% for women aged ≤34 years, 16% for woman aged 34-39 years and 28% for women aged ≥40 years (Table 4).

**Table 5:** Determinants of the number of patients undergoing Fresh Cycles

	**Base model and lag**				**With anticipatory behaviour and lag**			
				**all age**				**all age**
**Variable**	**Age ≤ 33**	**34 ≤ Age ≥ 39**	**Age ≥ 40**	**groups**	**Age ≤ 33**	**34 ≤ Age ≥ 39**	**Age ≥ 40**	**groups**
**EMSNCap effect**	-259.55	-342.52***	-330.17***	-232.62***	-155.83*	-201.41***	-291.93***	-141.46**
	126.39	51.06	44.67	62.61	74.49	33.61	52.81	42.22
Lag of D. Variable	0.90***	0.33**	-0.10	0.77***	0.96***	0.60***	-0.04	0.86***
	0.12	0.11	0.14	0.10	0.06	0.08	0.16	0.06
Season 1 (base)								
Season 2	310.06**	113.54**	-34.50	104.95*	216.99***	62.26*	-51.00	34.32
	85.99	33.07	29.51	40.93	47.91	25.00	36.27	31.38
Season 3	325.46**	127.08***	-98.78**	90.78*	267.69***	80.25**	-112.13**	44.48
	88.17	31.28	28.66	43.82	59.22	23.80	33.19	35.47
Season 4	67.44	-14.72	-212.63***	-68.20	-23.64	-102.07***	-230.53***	-138.63***
	81.04	35.35	31.35	37.99	44.52	25.59	33.83	28.61
Time Trend	23.26*	30.18***	42.17***	20.51***	12.70	16.29***	38.20***	11.13**
	10.66	4.53	5.69	5.67	7.32	3.29	6.76	4.01
Constant	-185.66***	561.15***	917.83***	123.85	-130.18***	378.40***	896.18***	127.05***
	112.46	105.92	100.94	98.47	93.02	71.92	110.12	69.31
N	32	48	32	112	32	48	32	112
df	6	6	6	6	6	6	6	6
R squared	0.87	0.78	0.88	0.73	0.94	0.85	0.87	0.82

OLS Regression method is used. Estimated coefficients and standard errors are presented. df denotes the degrees of freedom. * Significance at 10%, ** Significance at 5%, *** Significance at 1%. Each column reports a separate model specification.

#### Distribution of the number of cycles undertaken in a year

**Figure 3:**
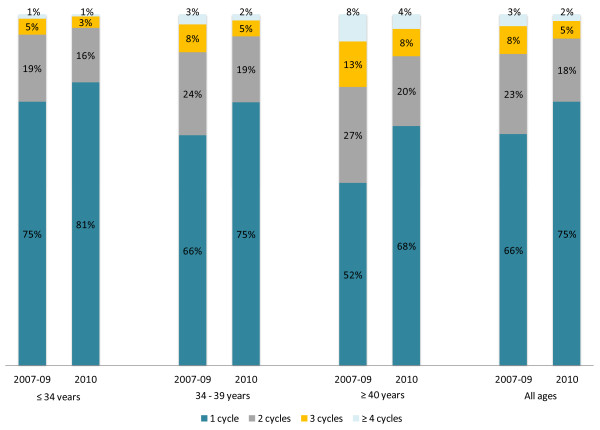
Percentage of patients undertaking 1 or more Fresh Cycles per year before and after the EMSNCap policy.

In terms of the number of cycles undertaken by individual women in a calendar year, all age groups undertook statistically significantly fewer Fresh Cycles after the EMSNCap policy (p < 0.05). For example, of women aged ≥40 year olds, 49% had more than 1 cycle a year before the policy, but this decreased to 33% of ≥40 year old women after the policy. This trend was seen in all age groups but was more pronounced in the older age groups (Figure 3). This indicates that of women who can afford to have at least one Fresh Cycle, they may not be able to afford the number of cycles they desire in a year. The clinical implication of this is that women may be delaying desired treatment, making them older and less likely to achieve a pregnancy, for example between 34 and 39 years each additional year of age decreases a women’s chance of a live birth following a Fresh Cycle by 9.6% [12].

#### Access to frozen cycles by age

**Figure 4:**
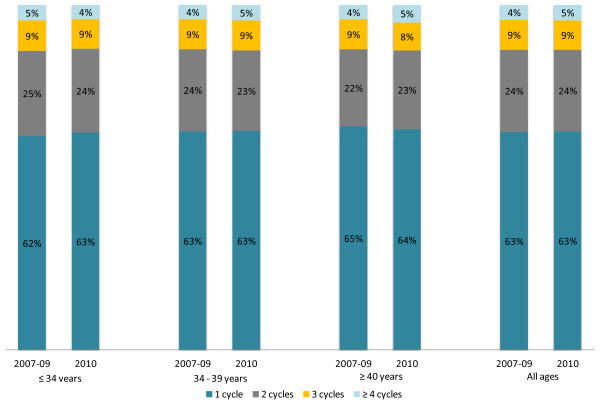
Percentage of patients undertaking 1 or more Frozen Cycles per year before and after the EMSNCap policy.

The econometric estimates of the effect of the EMSNCap policy on Frozen Cycles are summarised in Tables 6 and 7. In contrast to Fresh Cycles, the coefficient for the EMSNCaps policy was only statistically significant at the 10% level across all ages (-60 cycles quarter, 1700 cycles over 12 months) and for women age 34-39 years (-50 cycles per quarter). In percentage terms this represented an average decrease of 9% in Frozen Cycles due to the introduction of the EMSNCaps. After accounting for anticipatory behaviour, statistical significance only remained for women aged 34-39 years (Table 4). The distribution of the number of Frozen Cycles undertaken by women in a calendar year was not significantly different before or after the EMSNCaps (Figure 4).

**Table 6:** Determinants of number of Frozen Cycles undertaken

	**Base model and lag**				**With anticipatory behaviour and lag**			
				**all age**				**all age**
**Variable**	**Age ≤ 33**	**34 ≤ Age ≥ 39**	**Age ≥ 40**	**groups**	**Age ≤ 33**	**34 ≤ Age ≥ 39**	**Age ≥ 40**	**groups**
**EMSNCap effect**	**-90.14**	-50.24*	**-35.17**	-59.55*	**-38.81**	-58.57*	**55.71**	**-26.62**
	45.76	20.56	25.85	24.07	45.34	22.29	28.44	26.25
Lag of D. Variable	0.97***	0.62***	0.54*	0.92***	0.97***	0.59***	0.18	0.92***
	0.07	0.13	0.21	0.06	0.06	0.13	0.18	0.05
Season 1 (base)								
Season 2	79.41*	-81.01**	-108.96***	-69.09**	24.84	-72.15*	-99.61**	-102.30***
	37.06	28.25	28.95	21.47	41.84	28.55	33.33	23.52
Season 3	142.93**	-48.96*	-114.18***	-30.31	110.68*	-43.48	-105.86**	-50.03*
	48.55	23.59	22.17	23.94	49.01	23.81	28.91	24.67
Season 4	-13.55	-133.59***	-175.47***	-127.38***	-58.60	-125.68***	-193.05***	-155.11***
	37.56	25.04	22.98	21.83	37.75	25.33	27.67	22.09
Time Trend	7.94	7.03***	10.11***	6.05*	2.90	8.07*	6.77*	2.92
	4.18	3.09	2.58	2.45	3.79	3.16	2.34	2.38
Constant	-68.45***	308.07***	273.69**	83.05*	-4.29	320.05***	466.51***	127.48***
	65.82	71.06	78.96	34.55	59.81	72.13	69.14	37.82
N	32	48	32	112	32	48	32	112
df	6	6	6	6	6	6	6	6
R squared	0.92	0.71	0.87	0.87	0.92	0.69	0.88	0.85

OLS Regression method is used. Estimated coefficients and standard errors are presented. df denotes the degrees of freedom. * Significance at 10%, ** Significance at 5%, *** Significance at 1%. Each column reports a separate model specification.

**Table 7:** Determinants of the number of patients undergoing Frozen Cycles

	**Base model and lag**				**With anticipatory behaviour and lag**			
				**all age**				**all age**
**Variable**	**Age ≤ 33**	**34 ≤ Age ≥ 39**	**Age ≥ 40**	**groups**	**Age ≤ 33**	**34 ≤ Age ≥ 39**	**Age ≥ 40**	**groups**
**EMSNCap effect**	**-73.96**	-46.79*	**-36.78**	-53.04*	**-41.59**	-56.59***	**41.87**	**-27.31**
	40.07	17.34	22.78	20.93	36.48	19.98	24.30	23.57
Lag of D. Variable	0.97***	0.63***	0.51*	0.91***	0.97***	0.58***	0.24	0.90***
	0.07	0.11	0.20	0.06	0.06	0.13	0.17	0.06
Season 1 (base)								
Season 2	79.51*	-58.75**	-86.95**	-47.08*	45.19	-48.91*	-95.54**	-72.65***
	29.84	21.50	25.75	18.15	32.44	22.89	29.12	20.39
Season 3	131.32**	-34.06	-89.68***	-15.67	111.08**	-28.14	-94.70***	-30.90
	39.86	18.09	19.54	20.04	38.79	18.89	25.15	21.07
Season 4	33.76	-92.77***	-139.64***	-81.78***	5.10	-84.06***	-166.53***	-103.17***
	31.91	19.69	20.56	18.83	29.80	20.61	24.60	19.33
Time Trend	6.72	5.96*	9.12***	5.33*	3.50	7.22	5.12*	2.94
	3.62	2.47	2.22	2.11	3.09	2.66	2.06	2.08
Constant	-73.39***	249.38***	236.99***	61.64***	-35.46***	268.82***	387.39***	99.02**
	55.99	57.48	66.35	31.99	49.41	62.74	59.05	35.34
N	32	48	32	112	32	48	32	112
df	6	6	6	6	6	6	6	6
R squared	0.92	0.71	0.84	0.85	0.92	0.67	0.86	0.83

OLS Regression method is used. Estimated coefficients and standard errors are presented. df denotes the degrees of freedom. * Significance at 10%, ** Significance at 5%, *** Significance at 1%. Each column reports a separate model specification.

The contrasting findings of the impact of the EMSNCap policy on Fresh and Frozen Cycles is likely because Frozen Cycles are cheaper that Fresh Cycles, with average out-of-pocket expenses for a Fresh Cycle around $2600 compared to $1000 for a Frozen Cycle (Table 2). In addition, women who undertook Fresh Cycles prior to January 2010 - either in anticipation of the EMSNCaps or as planned treatment - would be likely to use up their frozen embryos in 2010 prior to discontinuing ART treatment or undertaking another Fresh Cycle. Therefore the impact of the policy with regard to Frozen Cycles may not become fully evident for some time.

#### Access to IUI cycles by age

Equation (1) was also used to estimate the effect of the EMSNCap policy on access to IUI Cycles. The estimates show that the EMSNCap policy had little impact on overall access to IUI treatment across all age groups. However, the number of IUI Cycles decreased significantly by 79 IUI Cycles per quarter (2200 cycles in 12 months) for women aged ≤34 years, representing a 14% reduction in cycles due to the EMSNCaps in this age group. This effect were eliminated after adjusting for anticipatory behaviour (Tables 4, 8 and 9). IUI is a less invasive and cheaper alternative to ART, with estimated out-of-pocket expenses of $650, which may explain why it was unaffected by the EMSNCaps. IUI is often used as a first-line treatment option for infertility before patient’s progress to ART, or as an alternative (substitute) to ART treatment. However, it has the disadvantages of lower success rates (approximately 10-15% per cycle, compared to 20-30% for ART) and less controllable means of minimising the risk of multiple births.

**Table 8:** Determinants of number of IUI Cycles undertaken

	**Base model and lag**				**With anticipatory behaviour and lag**			
				**all age**				**all age**
**Variable**	**Age ≤ 33**	**34 ≤ Age ≥ 39**	**Age ≥ 40**	**groups**	**Age ≤ 33**	**34 ≤ Age ≥ 39**	**Age ≥ 40**	**groups**
**EMSNCap effect**	-79.21**	**-22.85**	21.25*	**-59.55**	**3.36**	**-9.30**	**18.14**	**-1.43**
	27.87	13.25	10.88	24.07	38.94	16.75	15.93	15.81
Lag of D. Variable	-480.27***	256.94	64.32	0.92	0.99***	0.84***	0.14	0.97***
	16.30	22.18	23.48	0.06	0.04	0.07	0.20	0.03
Season 1 (base)								
Season 2	122.57***	-22.27	-8.17	45.30	73.13*	-35.75*	-5.55	-13.48
	24.83	13.27	8.69	59.14	33.54	13.54	14.71	13.22
Season 3	172.65***	-39.82	-26.86	45.98	14.63	-49.11***	-24.10	-42.19***
	24.91	13.04	8.86	63.19	29.16	13.30	14.96	11.54
Season 4	61.92**	-110.04**	-63.94***	-28.21	-120.60***	-121.66***	-61.02***	-117.94***
	20.97	12.92	8.08	56.94	29.85	14.76	12.17	11.89
Time Trend	-4.42	1.16	1.20	-0.83	-0.80	-0.27	1.43	-0.23
	2.82	1.70	1.23	8.28	3.90	1.85	1.59	1.62
Constant	993.48***	107.76***	155.42***	436.08***	15.93	114.26**	157.46***	54.97**
	33.95	34.26	12.46	94.98	48.47	36.48	27.73	18.33
N	32	48	32	112	32	48	32	112
df	6	6	6	6	6	6	6	6
R squared	0.98	0.84	0.82	0.85	0.95	0.83	0.70	0.96

OLS Regression method is used. Estimated coefficients and standard errors are presented. df denotes the degrees of freedom. * Significance at 10%, ** Significance at 5%, *** Significance at 1%. Each column reports separate model specification.

**Table 9:** Determinants of number of patients undergoing IUI Cycles

	**Base model and lag**				**With anticipatory behaviour and lag**			
				**all age**				**all age**
**Variable**	**Age ≤ 33**	**34 ≤ Age ≥ 39**	**Age ≥ 40**	**groups**	**Age ≤ 33**	**34 ≤ Age ≥ 39**	**Age ≥ 40**	**groups**
**EMSNCap effect**	**-14.44**	**-8.04**	19.87***	**-5.30**	**2.16**	**-4.36**	**17.49**	**0.37**
	24.76	8.24	7.10	9.20	25.65	8.99	9.13	9.39
Lag of D. Variable	0.99***	0.90***	0.25	0.98***	0.99***	0.89***	0.27	0.97***
	0.04	0.06	0.13	0.03	0.04	0.06	0.19	0.023
Season 1 (base)								
Season 2	57.51**	-19.46*	-8.81	-3.79	44.76	-23.11***	-8.61	-8.46
	19.11	7.28	8.48	7.96	21.82	7.64	8.56	8.07
Season 3	22.82	-30.52***	-20.25*	-21.89**	14.83	-32.58***	-20.43**	-24.77**
	19.56	7.17	8.01	7.48	20.40	7.61	8.62	7.50
Season 4	-49.43**	-71.08***	-40.98***	-63.29***	-61.90**	-74.04***	-40.29***	-67.68***
	17.29	7.54	6.45	7.05	18.38	7.82	7.13	7.13
Time Trend	0.90	0.45	0.88	0.38	-0.61	0.09	1.01	-0.14
	2.40	0.10	0.83	0.93	2.38	0.96	0.89	0.95
Constant	-10.06	52.81**	95.94***	25.99*	7.82	58.33**	92.27***	32.29**
	27.85	17.29	9.93	10.17	29.77	17.61	16.82	10.53
N	32	48	32	112	32	48	32	112
df	6	6	6	6	6	6	6	6
R squared	0.96	0.89	0.85	0.97	0.96	0.88	0.81	0.97

OLS Regression method is used. Estimated coefficients and standard errors are presented. df denotes the degrees of freedom. * Significance at 10%, ** Significance at 5%, *** Significance at 1%. Each column reports separate model specification.

#### Medicare benefits savings and number of cycles not performed due to policy

Table 10 summarises the savings in Medicare benefits to the Australian Government as a result of the EMSNCap policy. Using the econometric models to estimate the difference in the predicted and observed Medicare benefits paid, it was estimated the $84.2 million was saved in the first year after the EMSNCaps ($76.2 million after adjusting for anticipatory behaviour). Further, almost 6900 ART cycles were not undertaken in 2010 (5426 after adjusting for anticipatory behaviour) as a result of the EMSNCap policy, equating to approximately 1540 ART babies not being born in 2010 (1209 after adjusting for anticipatory behaviour).

**Table 10:** Savings in Medicare benefits, and the number of ART children not born in 2010 as a result of the EMSNCap policy

		**Without adjustment for anticipatory behaviour**		**With adjustment for anticipatory behaviour**	
	**Average**				
	**Medicare**				
	**benefit**		**Total**		**Total**
**Fresh Cycles**	**per cycle**	**Cycles**	**savings**	**Cycles**	**savings**
Predicted benefits and reduction in cycles - without policy:	$6199	5451	$33,790,749	4017	$24,901,383
Observed benefits and number of cycles - with policy:	$5108	31,504			$32,341
Savings due to policy	($1091)	31,504	$34,366,787	32,341	$35,279,845
**Total savings Fresh Cycles**			**$68,157,536**		**$60,181,228**
Live-birth rate per Fresh Cycle		21%		21%	
ART live-births not born due to policy		1145		844	
ART Multiple-birth rate		10.2%		10.2%	
ART infants not born due to policy		1261		930	
Frozen Cycles *					
Predicted Avr benefits and reduction in cycles without policy:	$1638	1409	$2,307,942	1,409	$2,307,942
Actual benefits and number of cycles with policy:	$1348	21,276		21,276	
Savings due to policy	($290)	21,276	$6,171,063	21,276	$6,171,065
**Total savings Frozen Cycles**			**$8,479,005**		**$8,479,005**
Live-birth rate per Frozen Cycle		18%		18%	
ART live-births not born due to policy		254		254	
Multiple-birth rate		10.2%		10.2%	
ART infants not born due to policy		279		279	
IUI Cycles *^+^					
Predicted Avr benefits and reduction in cycles without policy:	$1270				
Observed benefits and number of cycles with policy:	$549	10,504		10,504	
Savings due to policy	($721)	10,504	$7,571,632	10,504	$7,571,632
**Total savings IUI Cycles**			**$7,571,632**		**$7,571,632**
**Total Savings in Medicare benefits**			**$84,208,172**		**$76,231,865**
**Total ART infants not born due to policy**			**1540**		**1209**

All dollar figures in constant 2010 Australian dollars. * Anticipatory behaviour was not observed for Frozen and IUI Cycles. ^+^ IUI cycles were not affected by the EMSNCap policy.

## Discussion

This paper used econometric models to evaluate the introduction of a policy change in January 2010 which capped the amount the Australian Government pays in benefits for ART and IUI treatment cycles. In effect this policy removed the government’s exposure to increases in provider fees and strengthened price signals to patients. Because fees remained relatively stable after the introduction of the EMSNCaps, the out-of-pocket costs to patients for ART and IUI treatment increased on average by $500-$1000 for Fresh Cycles, and $300-$500 for Frozen Cycles and IUI Cycles. For Fresh Cycles, this resulted in a significant reduction in cycles across all age groups. After accounting for anticipatory behaviour - where women brought forward their treatment to 2009 to avoid the price rise - the policy reduced the number of Fresh Cycles in the 15 months after the policy by 16%. The impact of the policy was particularly evident in the older age groups; with an average reduction of 14% for women aged ≤34 years (10% significance), 21% reduction for woman aged 34-39 years (1% significance) and 38% reduction for women aged ≥40 years (1% significance). In contrast the policy did not have a significant impact on utilisation of Frozen Cycles after adjusting for anticipatory behaviour, nor did it have a significant impact of IUI cycles. These results were reflected in the distribution of women having one, two, three or four or more cycles per year during the pre and post EMSNCap periods. Therefore, not only did fewer women access Fresh Cycles after the policy change, but those who did underwent fewer Fresh Cycles in 2010. Further research is needed to elucidate why the policy differentially affected access to treatment by older women who conceivable could have more economic resources to fund fertility treatment than younger women.

While the data indicated that Frozen Cycles, which are substantially cheaper than Fresh Cycles, were not affected by the policy, this may be a temporary phenomenon. A Frozen Cycle can only proceed a Fresh Cycle that created the embryos to be transferred, therefore it is likely that women used up their stored Frozen embryos in 2010 and may not return for subsequent Fresh Cycles. This is supported by the high degree of anticipatory behaviour observed for Fresh Cycles in late 2009.

A limitation of this study was that the evaluation was restricted to 15 months after the introduction of the policy change. It remains to be seen if the utilisation rates and growth return to pre policy levels. An analysis of the introduction of co-payments from ‘no-fees’ to €1500-2000 for Fresh Cycles in Germany in 2004 found a 53% reduction in cycles in the first year after the policy [13]. Analysis of more recent German registry data indicates that utilisation for Fresh Cycles remains at 50% of 2003 levels in 2006 [3]. The German policy analysis also found that demand for ovulation induction therapy remained independent of the demand for ART treatments which is consistent with our findings with respect to demand for IUI. For some women ARTs are their only chance for achieving a pregnancy, and most women will undertake less invasive and expensive treatments, such as ovulation induction and IUI, before resorting to ART.

The estimated savings to the government in the first year after the policy was introduced was $84.2 million ($76.2 after adjusting for anticipatory behaviour), more than the $69.4 million estimated on behalf of the government [10]. These savings accounted for only 0.47% of Medicare benefits paid in 2009 [14]. While the savings in Medicare benefits estimated in this policy evaluation are not dissimilar to those projected on behalf of the Australian Government, the later analysis did not investigate the age-specific impact of the policy, use econometric methods to look at the impact of the policy on uptake of different cycle types, and did not estimate the number of ART children not born as a consequence of the policy.

In terms of access to treatment, conservatively 16% of women who were able to afford ART treatment in 2009, could not afford to undertake the treatment in 2010. This translated into between 1200 and 1500 ART babies not being born in 2010 in Australia. When these results were announced in November 2011, it caused considerable media coverage and debate [15,16]. Evaluating ART treatment using purely traditional health economic methods, such at cost-effectiveness and cost-utility analyses, is problematic because ARTs, unlike other healthcare interventions, are judged by their ability to create life rather than to extend or improve quality of life. This creates challenges for health economists and policy makers when making decisions about healthcare resourcing. An alternate method of valuing the worth of ART treatment in economic terms is to consider ART treatment as an investment from a government accounts perspective. One study that took this approach found that ART treatment represents an 8-fold return on investment based on the net future tax revenues of ART conceived children [17]. Due to these methodological challenges and strong sociocultural norms associated with ARTs, they are often targeted for funding cuts or increases as evidenced by the frequent changes in funding in Australia and throughout Europe [8].

It should be recognised that it is not only the infertile couple that suffers when funding is reduced but the quality of ART clinical practice, which ultimately negatively impacts the health outcomes of ART children. A number of studies in the United States [18-22] and Australia [23], have shown that when ART treatment costs increase, not only is equity of access reduced, but a financial incentive is created to transfer multiple embryos during treatment, thereby increasing the chance of a pregnancy in one cycle (i.e. it is costly to fail treatment and pay for another cycle). For example, in countries with supportive funding for ART such as the Nordic Countries and Australia the percentage of cycles where one embryo is transferred during treatment (single embryo transfer), is over 65%. This is in stark contrast to 15% of cycles in the UK which has very limited public funding for ART, and less than 12% of cycles in the United States which has no public funding for ART [3,5]. Therefore, less affordable treatment can lead to increased rates of twins and triplets, who have significantly higher risks of preterm birth, low birth rates, cerebral palsy and long term health problems [24-26]. A recent study from Australia showed that the positive trend to single embryo transfer from 29.5% of cycles in 2002 to 67.7% of cycles in 2008 resulted in the reduction in the multiple birth rates from 18.8% to 8.6% of ART births over the same period. The savings in birth-admission costs alone to the Australian Government was $48 million, theoretically funding over 50% of the increase in ART utilisation since 2002 and the birth of 2800 ART babies [23].

## Conclusions

This policy evaluation of recent changes to public funding for ART in Australia found that access to Fresh Cycles was significantly reduced for all age groups. With demand for ART treatment likely to increase due to the trend later childbearing, growing rates of obesity and some sexually transmitted diseases [27], it is important that changes to ART funding consider not only equitable access to treatment but its impact on clinical practice and the welfare of ART children and their families.

## Competing interests

Dr Georgina M Chambers is an Australian Postdoctoral Research Fellow (Industry). Grant No: LP100200165. Principle Organisation: Australian Government, Australian Research Council (ARC). Partner Organisations: IVFAustralia, Melbourne IVF, Queensland Fertility Group. Mr Van Phuong Hoang is currently employed as an Economics Research Officer on the same Grant. Dr Rong Zhu was employed as a Economics Research Officer on the same grant during the initial analysis period, and is now employed by Flinders University as an Economics Research Fellow. Associate Professor Peter J Illingworth is Medical Director of IVFAustralia and a shareholder of Virtus Health (IVFAustralia is part of the Virtus Health Network).

## Authors’ contributions

GMC was responsible for the study concept and design, data acquisition, methods development, and drafting a revising the manuscript. VPH participated in the methods development, data analysis and interpretation, and drafting the manuscript. RZ participated in the methods development, data analysis and interpretation, and drafting the manuscript. PJI participated in the study concept and design, interpretation of results and revising the manuscript. All authors read and approved the final manuscript.

## Pre-publication history

The pre-publication history for this paper can be accessed here:

http://www.biomedcentral.com/1472-6963/12/142/prepub
